# Supplementary material for: Vesicular Integral-Membrane Protein 36 Is Involved in the Selective Secretion of Fucosylated Proteins into Bile Duct-like Structures in HepG2 Cells
Source: Int J Mol Sci. 2023 Apr 11;24(8):7037. doi: 10.3390/ijms24087037 (PMC10138374; doi:10.3390/ijms24087037)
Supplement: Supplementary file 1 [file ijms-24-07037-s001.zip › ijms-2281047-supplementary.pdf]

(A)

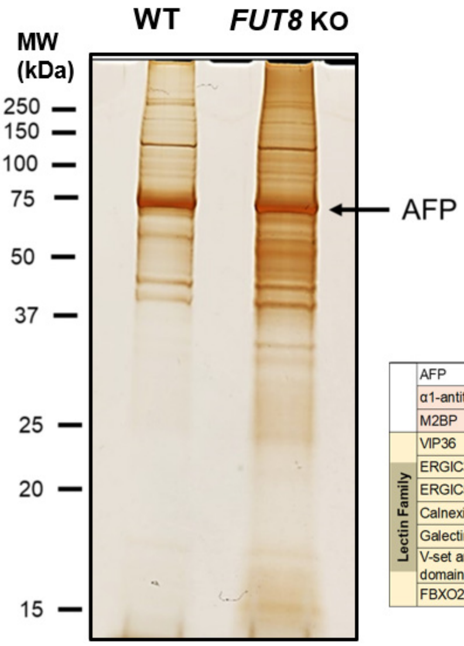

(B)

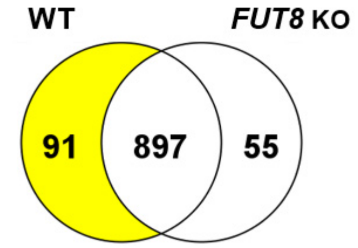

(C)

		WT	*FUT8* KO
	AFP	1630	1891
	α1-antitrypsin	14	0
	M2BP	4	2
Lectin Family	VIP36	2	6
Lectin Family	ERGIC32	3	1
Lectin Family	ERGIC53	5	2
Lectin Family	Calnexin	8	3
Lectin Family	Galectin-3	7	0
Lectin Family	V-set and immunoglobulin domain-containing protein 8	6	0
Lectin Family	FBXO2	6	4

		WT	*FUT8* KO
BC	Major vault protein (MVP)	12	0
BC	Aminopeptidase N (CD13)	11	3
BC	RACK1	14	9
Others	Transitional endoplasmic reticulum ATPase (VCP)	41	28
Others	Secretory carrier-associated membrane protein 3 (SCAMP3)	2	0
Others	SEC24C	19	7
Others	F11R (JAM-A)	5	1
Others	Glypican-3	3	0

Supplemental Figure S1. Identification of intracellular binding molecules involved in AFP transport. We aimed to identify the cargo receptor of AFP using the Strep-tag/Strep-Tactin system. HepG2 cell lysates that expressed AFP-Strep-tag were reacted with Strep-Tactin beads, and the sediment was electrophoresed using a 10% SDS-acrylamide gel. (A) Silver-stained gel. (B) Results of mass spectrometry as a Venn diagram. (C) Classification of the proteins identified by mass spectrometry. The numbers represent the number of hits for each protein. BC: bile canaliculus (bile duct-like structure).
